# Altered frontotemporal glucose metabolism following radiotherapy and/or chemotherapy for cervical cancer

**DOI:** 10.3389/fneur.2025.1598913

**Published:** 2025-06-19

**Authors:** Yao Hu, Yuan Zhong, Yuxiao Hu

**Affiliations:** ^1^Department of PET/CT Center, Jiangsu Cancer Hospital and Jiangsu Institute of Cancer Research and the Affiliated Cancer Hospital of Nanjing Medical University, Nanjing, China; ^2^School of Psychology, Nanjing Normal University, Nanjing, China; ^3^Jiangsu Key Laboratory of Mental Health and Cognitive Science, School of Psychology, Nanjing Normal University, Nanjing, China; ^4^NHC Key Laboratory of Nuclear Medicine and Jiangsu Key Laboratory of Molecular Nuclear Medicine, Wuxi, China

**Keywords:** cervical cancer, brain FDG PET, radiotherapy, chemotherapy, radiochemotherapy

## Abstract

**Background:**

Little is known about the effects of subphrenic radiotherapy on brain glucose metabolism in patients with cervical cancer (CC) after chemotherapy. This study aimed to explore the effects of radiotherapy, chemotherapy, and radiochemotherapy on brain glucose metabolism in patients with CC.

**Methods:**

A total of 237 CC patients who underwent ^18^F-fluorodeoxyglucose (^18^F-FDG) positron-emission tomography (PET)/computed tomography (CT) were included, consisting of 88 patients without treatment, 61 patients with radiotherapy, 24 patients with chemotherapy and 64 patients with radiochemotherapy. One-way analysis of variance (ANOVA) was used to explore the effects of chemotherapy, radiotherapy and radiochemotherapy factors on brain PET data in CC patients by using statistical parametric mapping (SPM).

**Results:**

Compared to CC patients without treatment, hypometabolism in some frontal and temporal lobes and no hypermetabolic regions were observed in those with radiotherapy (*P_FWEc_* < 0.05), while no significant brain metabolic areas was found in those with chemotherapy. Some above hypometabolic regions identified in radiotherapy and some other hypometabolic regions were found in patients with radiochemotherapy relative to those without treatment (*P_FWEc_* < 0.05). In addition, comparing any two of the radiotherapy, chemotherapy and radiochemotherapy groups only found significantly altered brain metabolic regions located in the right lingual gyrus between the radiotherapy and chemotherapy groups (*P_FWEc_* < 0.05).

**Conclusion:**

Radiotherapy might decrease metabolism in the temporal and frontal lobes in CC patients. Furthermore, chemotherapy and radiotherapy might synergistically decrease glucose metabolism in some frontotemporal regions of CC patients, which might indicate potential cognitive impairment and emotional disorders.

## 1 Introduction

Cervical cancer (CC) is the fourth most common malignant tumor in women (1). Due to advances in diagnosis and treatment methods (2), the 5-year survival rate of cervical cancer patients in China is as high as 60% (3). Thus, increasing attention is directed to the impact of treatment methods on the overall daily functioning of cervical cancer survivors, such as brain function (4). Because the underlying mechanism of brain dysfunction affected by treatments for noncentral nervous system cancers remains to be further studied, understanding of the effects of different treatments for peripheral tissue cancers is still limited.

Some previous studies have confirmed that up to 75% of patients have brain dysfunction during the process of systemic chemotherapy, and up to 60% of survivors show further brain damage after the completion of systemic chemotherapy (5–9). Research on brain ^18^F-FDG PET imaging can be adopted to study the underlying mechanism of altered brain function in patients with breast cancer, lymphoma and other cancers after systemic chemotherapy (10–15). Glucose, as the main metabolic substrate of the brain, produces sufficient energy through oxidation for brain activity. Thus, the local glucose metabolism of the different brain regions could be described by the local uptake of the glucose analog ^18^F-FDG. Brain ^18^F-FDG PET imaging may identify affected brain glucose metabolism under various conditions, which may help to examine mild differences in cognitive impairment even without significant structural damage (16). It provides a fine examination of treatment-related neurological changes, which may be better used to understand the neurological side effects in patients with cancer after different treatments.

Systemic chemotherapy has been demonstrated to reduce brain glucose metabolism in the cortical structures, deep nuclei, hippocampi, and cerebellum of patients with noncentral nervous system cancers (17, 18). One prior study in breast cancer found decreased frontal and increased parietal and insular metabolism exposure to chemotherapy (10). Other studies have also described reduced metabolic areas in the bilateral orbital frontal regions in patients with chemotherapy in comparison with control subjects (12) and significantly altered cerebral blood flow in the frontal cortex and cerebellum compared to those without treatment (11). Most studies have demonstrated that systemic chemotherapy has an impact on changes in regional brain metabolism in patients with different cancers, while there are no studies on CC. In addition, the treatment methods for CC patients include radiotherapy, chemotherapy, synchronous radiochemotherapy, and surgery, and radiochemotherapy is the most important therapy for most CC patients (2). However, most previous studies have mainly focused on the effects of systemic chemotherapy on brain glucose metabolism in patients with noncentral nervous system cancer (17) and the direct damage of brain radiation to brain function (19). Little is known about the effects of subphrenic radiotherapy on brain glucose metabolism and the interaction between subphrenic radiotherapy and systemic chemotherapy on brain metabolism in CC patients. With the purpose of exploring the effect of radiotherapy or the interaction between radiotherapy and chemotherapy on regional brain glucose metabolism in CC patients, this study analyzed the changes in brain glucose metabolism after exposure to radiotherapy and/or chemotherapy in CC patients by using brain resting-state ^18^F-FDG PET/CT. On the basis of some prior studies about altered brain metabolism after systemic chemotherapy for patients with other cancers, it was hypothesized that CC patients exposed to radiotherapy and/or chemotherapy would show altered regional brain glucose metabolism. It was also predicted that chemotherapy and radiotherapy might synergistically affect regional brain glucose metabolism in CC patients.

## 2 Methods

### 2.1 Subjects

In this study, a total of 237 patients with CC from Jiangsu Cancer Hospital were included. The brain PET images of all the subjects were gathered between January 2017 and January 2021. The inclusion criteria were as follows: (a): diagnosis of squamous cell carcinoma of the cervix, (b) ^18^F-FDG PET/CT scanning, (c) within half a year post-therapy, (d) right-handed subjects, (e) no neurological, psychiatric or mood disorders before diagnosis of cervical cancer, (f) no other oncology, (g) absence of brain metastasis, brain trauma, brain radiation and so on, (h) absence of suffering liver or renal disease, pregnancy or breastfeeding, and (i) images without defects or artifacts in visual evaluation. Clinical characteristics such as age, weight, height, diabetes, hypertension, surgery, radiotherapy, chemotherapy, International Federation of Gynecology and Obstetrics (FIGO) stage prior to any treatment in all patients with CC and brain PET images of all subjects were collected. Three hundred thirty-one patients included 88 patients without treatment, 77 patients with radiotherapy, 80 patients with chemotherapy and 86 patients with radiochemotherapy. Excluding subjects who underwent surgical treatment, a total of 237 subjects included 88 without treatment, 61 patients with radiotherapy, 24 with chemotherapy and 64 with radiochemotherapy were finally included in the present study.

### 2.2 Brain PET acquisition and preprocessing

All brain PET images of all subjects were acquired by using brain scanning with a three-dimensional (3D)-mode standard technique of PET/CT (Discovery 710, General Electric Medical Systems, Milwaukee, WI, United States). All patients fasted for at least 6 h before PET/CT examination, and the blood glucose level was below 11.1 mmol/L. PET/CT scanning was performed 50–70 min after intravenous injection of approximately 3.7–7.4 MBq/kg ^18^F-FDG. All subjects underwent noncontrast brain CT scan (300 mA; 120 kV) for anatomic localization, followed by brain PET scan, which was performed by using a 3D-mode standard technique in a 192 × 192 matrix with one bed position (5 min/bed position). The brain CT images were used for brain PET image attenuation correction. The brain PET images were reconstructed by using the ordered subsets expectation maximization algorithm.

All brain FDG images were preprocessed by using Statistical Parametric Mapping (SPM12, Wellcome Department of Cognitive Neurology, London, UK) in MATLAB 2013b (Mathworks, Natick, Massachusetts, United States). All brain images as DICOM files were converted to analyze files (NifTI format) for further analysis. The brain PET images of all subjects were normalized into the Montreal Neurological Institute space (MNI) (bounding box: −90, −126, −72; 90, 90, 108). The dimension and voxel size of the normalized brain PET images were 91 × 109 × 91 and 2 mm × 2 mm × 2 mm, respectively. The normalized brain PET images were smoothed by using a 10 mm full width at half maximum Gaussian kernel.

All subjects’ whole-body PET and CT images were transferred to the Beth Israel PET/CT viewer plugin for FIJI.^1^ The metabolic tumor volume (MTV) and total lesion glycolysis (TLG) for all tumor lesion of each patient were delineated semiautomatically with a ratio of 41% of the SUVmax value by FIJI. Small or low uptake tumor may be missed by the automatic segmentation, so we needed to add manually some volumes of interest.

### 2.3 Statistical analysis

Clinical data of all subjects were analyzed by one-way analysis of variance (ANOVA), or Kruskal-Wallis H Test, chi-square test and two-sample *t* test in SPSS 25.0 software (SPSS, IL, United States), and *p* < 0.05 was considered significant. Quantitative variables were expressed as the mean, range (normal distribution) or median, range (non normal distribution), and categorical variables were expressed as percentages. The brain PET images were analyzed by ANOVA to explore the effects of chemotherapy, radiotherapy and radiochemotherapy factors on CC patients by SPM. *Post hoc* analyses were used to evaluate the brain glucose metabolism differences between each pair of groups by the two-sample *t* test. Age was used as a covariate of no interest. A threshold of *p* < 0.05 family wise error correction (FWE) at the cluster level and with a threshold of *p* < 0.001 (uncorrected) was set in this study. The Talairach Client^2^ was used to convert the coordinates of these statistically significant points into corresponding anatomical locations in the Talairach atlas. The results of the SPM F/T maps were analyzed with MRIcroGL and BrainNet Viewer Toolbox to display the corresponding locations of significant metabolic clusters. The results are displayed with their individual cluster (voxels), F/T value, and MNI coordinates (x, y, z) of the peak voxels, brain regions, hemisphere and Brodmann area (BA).

## 3 Results

### 3.1 Clinical characteristics

Table 1 summarizes the characteristics of all the subjects in this study. There were significant differences among patients without treatment, with radiotherapy, with chemotherapy, and those with radiochemotherapy in age (*F* = 7.1, *p* = 0.000), MTV (*H* = 80.9, *p* = 0.000), TLG (*H* = 99.2, *p* = 0.000). Furthermore, there was not significant difference in MTV or TLG between patients with chemotherapy and those without treatment (*P_MTV_* = 1.000, *P_TLG_* = 1.000), neither was between radiochemothrapy and radiotherapy (*P_MTV_* = 0.733, *P_TLG_* = 0.320). The MTV or TLG in patiens with radiochemotherapy were significantly lower than those with chemotherapy (*P_MTV_* = 0.000, *P_TLG_* = 0.000) or those without treatment (*P_MTV_* = 0.000, *P_TLG_* = 0.000). The MTV or TLG in patients with radiotherapy were significantly lower than those with chemotherapy (*P_MTV_* = 0.000, *P_TLG_* = 0.000), and were lower than those without treatment (*P_MTV_* = 0.000, *P_TLG_* = 0.000). No significant differences were observed in BMI (*F* = 2.5, *p* = 0.061), hypertension (*χ^2^* = 4.2, *p* = 0.236), or diabetes (*χ^2^* = 1.8, *p* = 0.611), or pre-treatment FIGO stage (*H* = 4.8, *p* = 0.188). Significant differences were observed in chemotherapy cycles (*t* = 6.2, *p* = 0.000) between patients with chemotherapy and those with radiochemotherapy.

**Table 1:** Clinical and demographic characteristics.

	Without treatment (*n* = 88)	Radiotherapy (*n* = 61)	Chemotherapy (*n* = 24)	Radiochemotherapy (*n* = 64)	*P*
Age (mean, range)	51.9 (28–75)	57.8 (37–75)	52.6 (30–71)	50.7 (28–71)	0.000 (*F* = 7.1)
BMI (kg/m^2^) (mean, range)	24.6 (17.8–31.6)	23.9 (16.4–35.9)	23.6 (17.3–29.7)	23.1 (15.8–33.6)	0.061 (*F* = 2.5)
Hypertension [*n* (%)]	11 (12.5)	11 (18.0)	3 (12.5)	4 (6.3)	0.236 (*χ^2^* = 4.2)
Diabetes [*n* (%)]	5 (5.7)	5 (8.2)	2 (8.3)	2 (3.1)	0.611 (*χ^2^* = 1.8)
Chemotherapy cycles	–	–	1.92 (1–5)	3.8 (1–6)	0.000 (*t* = 6.2)
MTV (cm^3^) (median, range)	46.8 (0.0–454.5)	10.0 (0.00–201.2)a	46.2 (2.3–170.4)b	6.7 (0.0–108.5)ac	0.000 (*H* = 80.9)
TLG (g) (median, range)	376.0 (0.0–2567.4)	56.7 (0.00–1323.9)a	337.0 (7.4–2260.0)b	22.0 (0.0–728.1)ac	0.000 (*H* = 99.2)
Pre-treatment FIGO stage					
I	1	0	0	0	0.188 (*H* = 4.8)
II	6	9	1	7	
III	56	41	18	47	
IV	25	11	5	10	

BMI, body mass index; MTV, metabolic tumor volume; TLG, total lesion glycolysis; FIGO, International Federation of Gynecology and Obstetrics; a: compared with patients without treatment, *P* < 0.05, b: compared with patients with radiotherapy, *P* < 0.05, c: compared with patients with chemotherapy, *P* < 0.05.

### 3.2 Altered brain regions among patients without treatment, with radiotherapy, chemotherapy and radiochemotherapy

**Figure 1:**
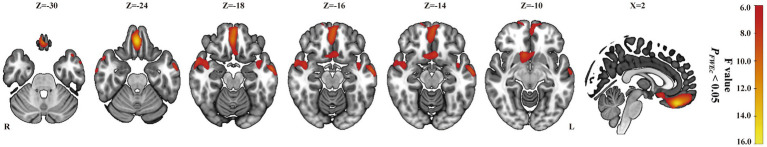
ANOVA in all subjects. Brain mapping depicting the combined effects of radiotherapy, chemotherapy and radiochemotherapy in one-way ANOVA (*P_FWEc_* < 0.05) located in the right rectal gyrus, right anterior cingulate, left subcallosal gyrus, and bilateral inferior, bilateral superior, right middle temporal gyrus.

**Figure 2:**
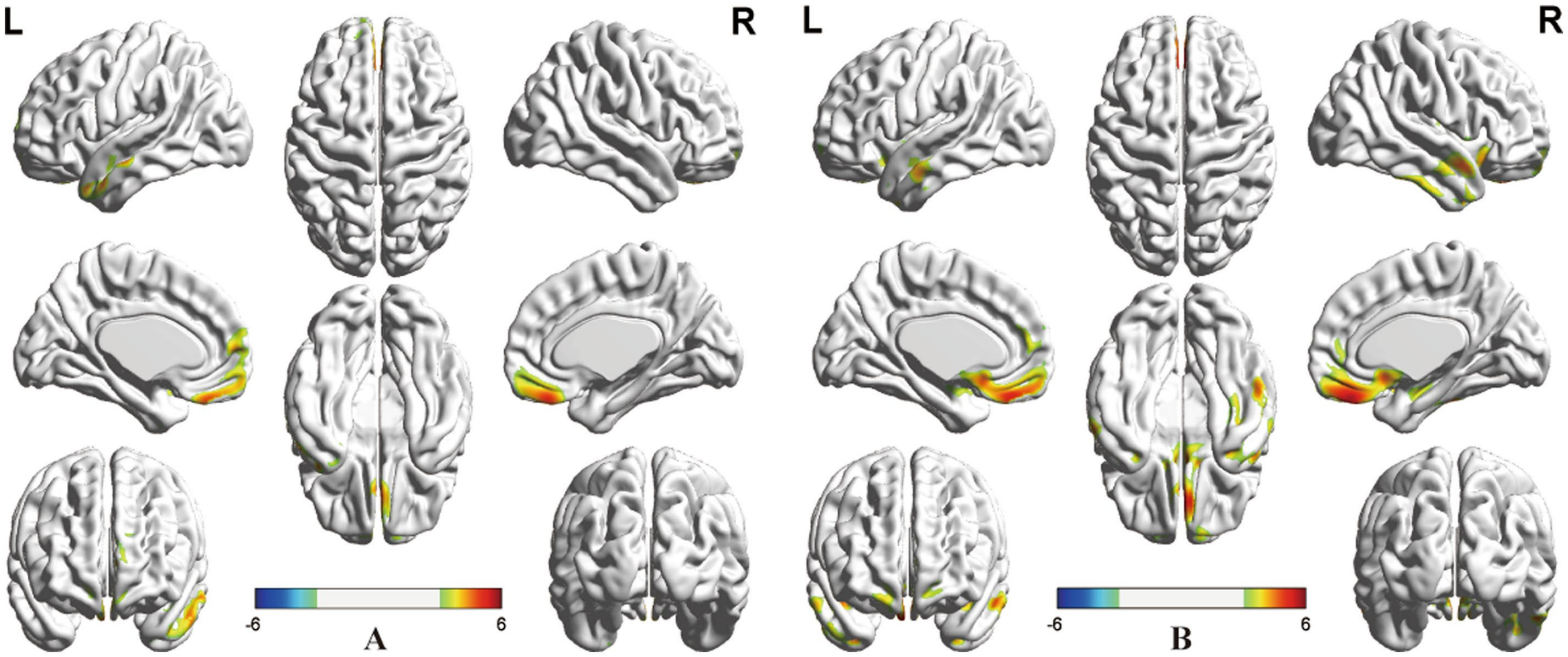
*Post-hoc* analysis. **(A)** Significant clusters with the effects of radiotherapy located in the right rectal and left medial frontal gyrus, and left middle and superior temporal gyrus. **(B)** Significant cluster with the effects of radiochemotherapy located in the right rectal gyrus, right anterior cingulate, and right middle and left superior temporal gyrus, and right fusiform gyrus (positive: radiotherapy or radiochemotherapy < without treatment, negative: radiotherapy or radiochemotherapy > without treatment, *P_FWEc_* < 0.05).

As shown in Table 2 and Figure 1, ANOVA revealed some significant brain metabolic differences, which were located in the right rectal gyrus (the largest cluster, BA11), right anterior cingulate (BA25), left subcallosal gyrus (BA25), bilateral temporal gyrus (BA21, 38). Compared with CC patients without treatment, those with radiotherapy showed hypometabolism in the right rectal gyrus (the largest cluster, BA11), left medial frontal gyrus (BA10, 11) and left middle and superior temporal gyrus (BA21, 38) (Table 2; Figure 2A), and those with radiochemotherapy showed hypometabolism in the right rectal gyrus (BA11), right anterior cingualate (BA25), left superior (BA22) and right middle (BA21) temporal gyrus and right fusiform gyrus (BA20) (Table 2; Figure 2B). No brain hypermetabolic areas were found in patients with radiotherapy, or radiochemotherapy relative to those without treatment. The results also showed that compared to patients with radiotherapy, those with chemotherapy exhibited hypometabolism in the right lingual gyrus (BA18) and no brain hypermetabolic areas (Table 3). In addition, there were no significant brain metabolic areas between the CC patients with chemotherapy and those with radiochemotherapy or those without treatment.

**Table 2:** Altered brain regions between patients without treatment and those with radiotherapy, chemotherapy or radiochemotherapy.

Brain regions	Hemisphere	BA	Voxels	*P_FWE-corr_*	F/T value (peak)	MNI coordinates (peak) x, y, z		
Altered brain regions								
Rectal gyrus	R	11	2,212	0.000	14.06	2	38	−24
Anterior cingulate	R	25			8.91	10	16	−10
Subcallosal gyrus	L	25			8.07	−4	16	−14
Inferior temporal gyrus	L	21	690	0.004	9.97	−60	−6	−18
Superior temporal gyrus	L	38			7.68	−44	16	−30
Superior temporal gyrus	L	38			6.91	−38	4	−18
Middle temporal gyrus	R	21	508	0.016	8.38	56	6	−18
Superior temporal gyrus	R	38			7.86	44	0	−16
Inferior temporal gyrus	R	21			7.19	64	−8	−24
Without treatment > radiotherapy								
Rectal gyrus	R	11	2027	0.000	−5.08	2	38	−24
Medial frontal gyrus	L	10			−4.41	−6	56	8
Medial frontal gyrus	L	11			−4.21	−4	60	−14
Middle temporal gyrus	L	21	938	0.001	−4.68	−56	4	−28
Middle temporal gyrus	L	21			−4.58	−64	−12	−12
Superior temporal gyrus	L	38			−4.44	−42	18	−32
Without treatment < radiotherapy								
*No suprathreshold clusters*								
Without treatment vs chemotherapy								
*No suprathreshold clusters*								
Without treatment > radiochemotherapy								
Rectal gyrus	R	11	7,110	0.000	−5.69	2	38	−24
Anterior cingulate	R	25			−5.08	8	14	−12
Superior temporal gyrus	L	22			−4.95	−60	−7	8
Middle temporal gyrus	R	21	3,382	0.000	−4.89	56	6	−18
Middle temporal gyrus	R	21			−4.79	34	4	−40
Fusiform gyrus	R	20			−4.72	56	−32	−26
Without treatment < radiochemotherapy								
*No suprathreshold clusters*								

BA, Brodmann area; MNI Coordinates, Montreal Neurological Institute Coordinates.

**Table 3:** Altered brain regions between any two groups from radiotherapy, chemotherapy and radiochemotherapy.

Brain regions	Hemisphere	BA	Voxels	*P _FWE-corr_*	T value (peak)	MNI coordinates (peak) x, y, z		
Radiotherapy > chemotherapy								
Lingual gyrus	R	18	702	0.016	−4.84	20	−84	−14
Lingual gyrus	R	18			−3.51	10	−64	2
Radiotherapy < chemotherapy								
*No suprathreshold clusters*								
Chemotherapy vs radiochemotherapy								
*No suprathreshold clusters*								
Radiotherapy vs radiochemotherapy								
*No suprathreshold clusters*								

## 4 Discussion

In this study, we explored the effects of radiotherapy and/or chemotherapy factors on brain glucose metabolism in CC patients. The findings showed that several different clusters of brain regional metabolism were affected by radiotherapy and/or chemotherapy factors. Our results suggested that chemotherapy and/or radiotherapy might decrease regional glucose metabolism in the CC patients, especially in the fronto-temporal regions.

Prior studies have demonstrated that chemotherapy could reduce brain glucose metabolism in patients with different cancers, such as breast cancer and lymphoma. However, in present study, no significant brain abnormally metabolic regions was found in patients received chemotherapy compared with those without treatment. The brain metabolic changes induced by chemotherapy were reported to be significantly associated with the number of chemotherapy cycles (20). The chemotherapy cycles in the group received chemotherapy were significantly fewer than those in the group received radiochemotherapy in this study. The reason for no significant brain metabolic changes might be due to fewer chemotherapy cycles. The brain abnormally metabolic regions in patients with radiochemotherapy were not completely consistent with those of patient received radiotherapy in present study. It means that chemotherapy might alter brain glucose metabolism in the frontal or temporal regions in CC patients. Silvermann et al. (11) found that the blood flow of the frontal regions was significantly altered in patients with breast cancer after chemotherapy through ^15^O-water PET imaging. In an another study by Chen et al. (21) found that reduced performance of attention networks task significantly correlated with increases in cerebral blood flow changes of some brain regions in patients with breast cancer. Their results suggested that chemotherapy might influence the blood flow in cerebral regions through increasing cerebral perfusion, which reduces the attention abilities in breast cancer patients. The hypometabolism in the frontal or temporal regions after chemotherapy in this study might be related to the local blood flow changes, which might be an attempt to restore homeostasis. The direct toxic impacts of chemotherapy on the brain are considered to be symmetrical, and asymmetric deviations may reflect the functional changes caused by treatment (22). In this study, hypometabolism mainly in asymmetric frontal or temporal lobes following chemotherapy in patients with CC might indicate potential dysfunction in these abnormal brain regions.

The rectal gyrus located on the medial surface of the frontal lobe, near the midline, and is part of the orbitofrontal cortex. It is a multifunctional cortical region that involved in higher-order cognitive and emotional regulatory processes. As a critical node in the frontal-limbic network, the rectal gyrus severs as a bridge for emotion, decision-making, olfaction, and social behavior. Ponto et al. demonstrated that patients with breast cancer after chemotherapy exhibited hypometabolism in the bilateral orbital frontal regions (12). Furthermore, their team also found that these breast cancer survivors had significantly lower scores in executive functioning, working memory and divided attention, which reflected potential functional impairment in frontal-subcortical brain regions (45). A prospective study based on functional MRI demonstrated that compared with the control group, the right medial frontal gyrus, right orbitofrontal cortex, right middle and bilateral superior temporal lobes, and left insular lobe showed low activation in patients with breast cancer after chemotherapy, indicating that chemotherapy played a role in brain functional differences (23). Additionally, Tashior et al. (24) demonstrated that reduced brain glucose metabolism located in the prefrontal lobe, anterior cingulate gyrus, lateral frontal gyrus, and insular cortex may be related to a depression state in patients without any treatment. Patients with CC reported more anxiety, dysphoria, anger and confusion than healthy controls (25). Brain functional abnormalities in the orbitofrontal cortex have also been reported in anxiety disorders in comparison with healthy controls (26). However, lacking of the evaluation of neuropsychiatric scales in this retrospective study cannot be fully revealed, but subclinical mental state might affect those regional brain metabolism. Hypometabolism in rectal gyrus or medial frontal gyrus might reflect bad abilities following chemotherapy and/or radiotherapy in patients with CC, it might exhibit a series of cognitive and emotional disorders (depression, obsessive-compulsive disorder and anxiety disorder).

The CC patients with radiotherapy also showed hypometabolism mainly in the right rectal gyrus, left medial frontal gyrus, and left middle and superior temporal gyrus compared to those without treatment. The middle and superior temporal gyrus is involved in language proceesing, visual motion perception, and social cognition and the superior gyrus is involved in auditory processing, language comprehension, and multimodal integration (27). Our results suggested that radiotherapy might mainly damage regional brain function in the bilateral frontal lobe and left temporal lobe. Pelvic radiotherapy for CC patients not only leads to pelvic radiation inflammation but also inhibits the proliferation of bone marrow cells in the irradiated area. Furthermore, some previous studies have found that subphrenic radiation therapy significantly increases inflammatory markers in patients (28). Subphrenic radiation could lead to gut dysbiosis, which will further result in gut leakage (29). Gut leakage facilitates intestinal microbe and/or compound infiltration into the bloodstream, increasing the systemic distribution of low inflammation and entering the brain. Therefore, all of the above pathways could ultimately promote the proliferation of glial cells and trigger neuroinflammation (30), which affects brain metabolism. Further research is needed to explore the effects of subphrenic radiotherapy on brain glucose metabolism in patients with different cancer.

To our knowledge, the interaction between chemotherapy and radiotherapy factors on brain glucose metabolism remains unclear. In this study, we found that the voxels and the largest cluster of altered regional brain glucose metabolism affected by radiochemotherapy were more and larger than those reduced by radiotherapy, and the abnormal brain glucose metabolic regions reduced by them were incompletely similar. Anterior cingulate hypometbolism was seen in radiochemotherapy and not in radiotherapy, while left medial frontal and left middle temporal hypometbolism were observed in radiotherapy and not in radiochemotherapy. The reason for these differences was that the results only list the mainly corresponding locations of significant metabolic clusters. The voxels of right anterior cingulate, right rectal, left superior temporal hypometabolism were more in patients received radiochemotherapy than those in patients received radiotherapy and right fusiform and right middle temporal hypometabolism were seen in radiochemotherapy, but not seen in radiotherapy. Anterior cingulate gyrus is involved in emotion processing, regulation to attention and cognitive control (31). It serves as a critical functional hub linking the limbic system to prefrontal brain regions. While, the fusiform gyrus is located at the bottom of the temporal lobe and is involved in high-level visual processing, integrating semantic, memory, and social cognitive functions. Those suggested that all the above hypometabolic brain regions might be related to the cognitive and mental state of the patients. And the cognitive and mental state of patients with radiochemotherapy might be worse than those with radiotherapy. The efficacy of radiotherapy partly relies on the activation of the immune system (32). Moreover, peripheral cytokines are increased in cancer survivors receiving different chemotherapeutic drugs, such as cisplatin and paclitaxel (33). The brain damage induced by systemic chemotherapy may be partially caused by proinflammatory cytokines. Therefore, radiotherapy and/or chemotherapy altered brain glucose metabolism in CC patients to some extent through immune activation, which might be a synergistic effect.

However, the voxels of left medial frontal and left middle temporal hypometabolism were fewer in radiochemtherapy than those in radiotherapy. In a previous longitudinal study on gray matter density, McDonald et al. (34, 35) reported that compared with healthy controls or the no chemotherapy group, a significant decrease in gray matter density in the frontal, temporal lobe at 1 month post chemotherapy did not completely recover at 1 year post chemotherapy. In another study using functional MRI, McDonald et al. (36) found that decreased activation in the frontal lobe at 1 month post chemotherapy recovered to pre-treatment state at 1 year post-treatment. These findings suggested time-dependent alterations in brain function or gray matter density. In this study, the treatment time in CC patients received radiochemotherapy was longer than those received radiotherapy. Hence, the reason for these differences might be related to the treatment time.

Our findings also found that chemotherapy might decrease glucose metabolism in the right occipital lobe (right lingual gyrus) in CC patients compared with radiotherapy. The occipital lobes connect with other brain parts through neurons, which participate in visuospatial recognition. The lingual gyrus, located in the medial of occipital lobe, serves as a multifunctional hub in visual processing, while also being closely associated with memory and emotional processing (37, 38). Regional brain hypometabolism in patients with Alzheimer’s disease frequently located in the occipital lobe is usually irreversible, which would mean that these brain areas are related to brain damage (39). However, hypometabolism in the occipital lobes was not found between patients with chemotherapy or radiotherapy and those without treatment in this study. Therefore, larger sample and prospective studies are needed to further elaborate the interactive effects of chemotherapy and radiotherapy on brain glucose metabolism in CC patients.

The patients’ tumor burden will relieved or disappear after receiving chemotherapy and/or radiotherapy. In this study, TLG and MTV of all lesions in the patient received radiotherapy or radiochemotherapy were fewer than those without treatment, but not found in patients received chemotherapy. Similar patterns also occurred in brain metabolic alteration of the frontal or temporal lobes between patients received radiotherapy or radiochemotherapy and those without treatment. Prior studies have demonstrated that TLG negatively correlated with glucose metabolism in some brain regions, such as some frontal, temporal, parietal regions, and positively correlated with other brain regions, such as the basal ganglia, insula, amygdala, and hippocampus in some untreated cancers (40, 41). Some other prior studies have reported that brain may regulate the growth and development of peripheral tumors through cancer information (42), which might be believed to reflect attempts to restore homeostasis (43). Radiotherapy or/and chemotherapy inhibit tumor growth and development lead to a reduction in tumor burden, which means that the transmission of cancer information through brain will decrease. In addition, there was no statistically significant difference in pre-treatment FIGO stage of all patients from different groups. Therefore, these brain hypometabolic areas in patients received radiotherapy or/and chemotherapy might be related to the tumor burden.

This study suggests that chemotherapy and radiotherapy might synergistically decrease glucose metabolism in the frontal and temporal lobes of CC patients, which may indicate potential cognitive impairment and emotional disorders. Therefore, the regular ^18^F-FDG PET/CT follow-up of patients receiving anticancer treatment may be extended to evaluate the changes in brain glucose metabolism caused by chemotherapy and/or radiotherapy. ^18^F-FDG PET/CT examination could not only provide valuable information for staging, curative effect and prognosis evaluation of cancer patients but also serve as an objective evaluation tool for individual brain glucose metabolism exposure to chemotherapy and/or peripheral radiotherapy.

## 5 Limitations

There are some shortcomings in this study. First, due to being a retrospective study, this study lacked the education level and neuropsychological testing of the CC patients. Second, radiotherapy and chemotherapy could lead to menopause or hormonal imbalances in female patients. The impacts of menopausal status or hormone levels on brain function are inconsistent in some studies (44). Menopausal status or hormone levels may affect the resting brain glucose metabolism of CC patients receiving therapies. Third, in this study, the number of chemotherapy cycles was different between chemotherapy and chemoradiotherapy. Chiaravalloti et al. found that alterations in brain regional metabolism may not be associated with chemotherapy cycles (13). Another study demonstrated that chemotherapy cycles may affect the brain metabolic changes induced by chemotherapy (20). In addition, this was conducted at a single center. Therefore, a multicenter and prospective study is needed to determine the role of menopausal status or hormone levels and other confounding factors and to provide more conclusive evidence for the effects of radiotherapy and/or chemotherapy on brain glucose metabolism in CC patients.

## 6 Conclusion

In the present study, radiotherapy might decrease metabolism in the temporal and frontal lobes in CC patients. Furthermore, chemotherapy and radiotherapy might synergistically decrease frontotemporal glucose metabolism in CC patients, which might indicate potential cognitive impairment and emotional disorders. In addition, brain FDG PET imaging might be expected to serve as an objective evaluation tool for abnormal brain metabolism in patients with cervical cancer after treatment.

## Data Availability

The original contributions presented in the study are included in the article/supplementary material, further inquiries can be directed to the corresponding author.

## Footnotes

^1^ http://sourceforge.net/projects/bifijiplugins/

^2^ http://www.talairach.org/manual.html
